# A study on corporate social responsibility of professional football clubs on fans’ consumption intentions in China

**DOI:** 10.3389/fphys.2025.1651356

**Published:** 2025-09-29

**Authors:** Chengfeng Zhang, Keke Song, Linhua Chen

**Affiliations:** ^1^ Department of Physical Education, Shanghai University of Engineering Science, Shanghai, China; ^2^ School of Sports Science and Engineering, East China University of Science and Technology, Shanghai, China

**Keywords:** professional football clubs, CSR, fans identification, motivational attribution, involvement, consumption intentions

## Abstract

**Purpose:**

To examine how Chinese fans’ perception of club CSR affects consumption intentions via fan identification and motivational attribution, and to test the moderating role of involvement.

**Methods:**

A total of 1,327 valid questionnaires were collected and analyzed using structural equation modeling and cluster analysis.

**Results:**

CSR perception had a significant positive effect on consumption intentions; this effect was fully mediated by fan identification and motivational attribution, with the model explaining 40.3% of the variance. Highly involved fans relied primarily on the fan identification path, whereas low-involvement fans relied on the motivational attribution path.

**Conclusion:**

Clubs should implement differentiated CSR strategies tailored to fans’ levels of involvement to effectively enhance consumption intentions.

## 1 Introduction

Previous studies have demonstrated that the fulfillment of corporate social responsibility (CSR, abbreviations are used hereafter) by professional sports clubs can positively influence fans’ consumption intentions (FCIs, abbreviations are used hereafter) (Walker and Kent, 2009; Lacey et al., 2015). However, to date all such studies have focused on European Professional Football Clubs, North American Professional Sports Franchises, and their fans. Furthermore, the development of CSR in Chinese professional football clubs has lagged behind that of their European and American counterparts. From the perspective of development trajectories, European and North American professional football clubs originated within local communities, and their supporters are members of a shared collective. Fulfilling social responsibility is therefore a voluntary, proactive act and a long-standing means of sustaining communal identity. Consequently, the history of social-responsibility engagement is extensive, its content and forms are rich and concrete, and supporters are exposed to a large volume of related information. By contrast, Chinese professional football clubs emerged from administrative restructuring and lack an inherent, proactive disposition toward social responsibility. Their engagement tends to be reactive, driven by external expectations, resulting in initiatives that are short-term and superficial. This creates a gap between supporters and CSR-related information, leading to markedly less exposure for Chinese fans. Consequently, Chinese fans’ perceptions of clubs’ CSR efforts likely differ significantly from those observed in Western contexts, raising questions about the direct applicability of conclusions drawn from international studies to the Chinese context.

Hence, our study examines whether the perceived CSR of professional football club influences FCIs in the Chinese context and explores the underlying mechanisms of this relationship. While Western studies have addressed similar questions, the dynamics remain underexplored in China due to distinct developmental trajectories of CSR initiatives in professional sports. To address this gap, we empirically analyze survey data from Chinese Super League (CSL) fans, focusing on how their perceptions of clubs’ CSR shape consumption behaviors.

## 2 Literature review and research hypotheses

Research indicates that proactive CSR initiatives by professional sports clubs generate strategic value, including enhanced consumer engagement through increased repurchase intentions and word-of-mouth advocacy (Walker and Kent, 2009; Lacey et al., 2015; Breitbarth and Harris, 2008). Previous studies on CSR perceptions and consumer behavior in this domain, predominantly grounded in organizational identity theory and relationship quality theory, have identified mediating mechanisms such as fan identification (FI, abbreviations are used hereafter), relationship quality (encompassing commitment, trust, and satisfaction), and emotional attachment (Jin, 2006; Walker and Kent, 2009; Scheinbaum and Lacey, 2015; Lacey and Kennett-Hensel, 2016). Western scholarship consistently demonstrates a positive association between CSR perceptions and consumer engagement, though these findings largely reflect contexts with mature CSR frameworks in professional sports.

Nevertheless, existing research has overlooked the role of motivational attribution (MA, abbreviations are used in the following text) in shaping fans’ responses to socially responsible consumption behaviors. Attribution theory posits that individuals’ perceptions and attitudes toward a behavior or event depend on their attribution of its underlying motives, such as self-interest versus altruism (Kelley, 1973; Weiner, 1992). Prior studies on CSR demonstrate that consumers’ attributions of CSR motives directly influence behavioral outcomes, including purchase intentions, word-of-mouth advocacy, brand loyalty, and reputation assessments (Mohr and Webb, 2005; Walker et al., 2010; Ellen et al., 2006; Groza et al., 2011). Consequently, MA — a psychological construct reflecting individuals’ evaluations of CSR motives — may serve as a critical mechanism mediating the relationship between fans’ perceptions of professional sports clubs’ CSR and their subsequent FCIs.

Furthermore, variations in fans’ involvement levels with professional football clubs may lead to divergent responses between highly involved (“hardcore”) and casually involved (“pseudo-fans”) subgroups regarding their perceptions of clubs’ CSR, identification with the club, attributions of CSR motives, and FCIs. To account for these differences, this study incorporates fan involvement as a moderating variable to examine its role in mediating the relationship between CSR perceptions and FCIs.

Building on this framework, the present research positions FI and MA as parallel mediating mechanisms while integrating involvement — a psychological characteristic reflecting fans’ engagement intensity — to investigate how Chinese fans’ CSR perceptions shape their FCIs. By testing this moderated mediation model, the study elucidates context-specific pathways through which CSR initiatives influence fan behavior in China’s evolving professional sports landscape.

### 2.1 Fans’ CSR perceptions and their FCIs

CSR initiatives positively influence consumers’ purchase intentions by aligning with their values and enhancing reputational, marketing, and public relations outcomes (Deng and Zhang, 2016; Ellen et al., 2006; Ma, 2011). These effects extend to professional sports, where empirical studies demonstrate that clubs’ CSR activities strengthen fans’ engagement and consumption intentions. For instance, Walker and Kent (2009) surveyed 297 National Football League (NFL) fans and revealed that perceptions of clubs’ CSR efforts significantly improved both club reputation and FCIs. Similarly, Lacey et al. (2015) examined North American professional basketball fans, revealing that CSR perceptions drive positive word-of-mouth advocacy. Building on this evidence, we propose the following hypothesis:

H1: The stronger fans perceive a professional football club’s CSR, the greater their FCIs.

### 2.2 The mediating role of FI

FI with professional sports clubs is reflected in distinct behavioral and psychological patterns. Highly identified fans actively seek club-related information, advocate for their teams within social networks (Ashforth and Mael, 1989), and use inclusive language (e.g., “How is our club performing?”) to signal psychological ownership. This identification further manifests through symbolic gestures such as wearing team-branded apparel and publicly signaling affiliation (Lock et al., 2012). During matches, strongly identified fans exhibit intense loyalty, engaging in coordinated cheering, disruptive tactics toward opposing players, and vocal criticism of referees’ contentious decisions (Cialdini et al., 1976). Extensive research demonstrates that fan identification generates strategic value for professional sports clubs, enhancing fan loyalty, strengthening brand equity, and increasing willingness to attend matches, purchase merchandise, and travel for away games (Wann et al., 2001; Sutton et al., 1997; Gladden and Funk, 2001). Within sports consumption research, scholars have empirically validated that clubs’ CSR initiatives positively influence FI (Gladden and Funk, 2001). Building on this evidence, we propose the following hypotheses:

H2: The stronger fans perceive a professional football club’s CSR, the stronger their identification with the club.

H3: The stronger fans’ identification with a professional football club, the greater their willingness to express FCIs.

H4: FI mediates the relationship between perceptions of a club’s CSR and FCIs.

### 2.3 Mediating role of MA

Attribution theory posits that individuals’ perceptions and attitudes toward behaviors or events depend on their causal inferences (Kelley, 1973). Consumers’ attributions of CSR motives are categorized as either altruistic (driven by social welfare goals) or self-interested (aimed at organizational gain) (Vlachos et al., 2013; Deng and Long, 2017). Consumers exhibit more favorable attitudes toward companies when they attribute CSR initiatives to altruistic motives rather than self-interest (Mason, 2001; Jiang and Shi, 2017). Empirical evidence confirms that CSR motive attributions directly influence consumer engagement, including purchase intentions, word-of-mouth advocacy, brand loyalty, and reputation evaluations (Walker et al., 2010). Furthermore, public perceptions of CSR intensity shape these attributions: higher perceived CSR levels correlate with more positive corporate evaluations (Gao, 2009). Klein and Dawar’s (2004) experimental study further substantiates this relationship, demonstrating that CSR intensity significantly predicts consumer attributions. Based on this framework, we propose the following hypotheses:

H5: The stronger fans perceive a professional football club’s CSR, the more likely they are to attribute its CSR efforts to altruistic motives.

H6: Fans who attribute CSR initiatives to altruistic motives demonstrate greater willingness to engage in FCIs.

H7: Altruistic attributions mediate the relationship between perceived CSR and FCIs.

### 2.4 MA and FI

Prior research indicates that fans exhibit positive identification with professional sports clubs’ CSR initiatives, which enhances their willingness to purchase, engage in word-of-mouth advocacy, and consume team merchandise (Walker and Kent, 2009). However, attribution theory suggests that such responses depend critically on fans’ causal inferences about clubs’ motives (Kelley, 1973; Weiner, 1989). Specifically, fans affirm CSR behaviors only when they attribute them to altruistic goals aimed at societal welfare. Conversely, if fans perceive CSR efforts as self-interested responses to external pressures or profit motives, their approval and engagement diminish significantly. This mechanism aligns with findings in marketing research: Kou (2008) demonstrated that consumers’ attributions of CSR motives directly shape their CSR identification. Building on this theoretical and empirical foundation, we propose:

H8: The stronger fans perceive altruistic motives underlying a professional football club’s CSR, the stronger their identification with the club.

### 2.5 Role of involvement influence

Involvement denotes an individual’s perceived relevance of a target object based on personal needs, interests, or values (Sherif and Cantril, 1947). In sports, fans represent a distinct consumer segment characterized by heightened involvement with specific sports or clubs (Shank et al., 1998; Underwood et al., 2001; Taylor and Rice, 2012). Scholarly work establishes that high fan involvement correlates strongly with attitudinal and behavioral loyalty, including game attendance, repurchase intent (i.e., repeated ticket purchases), and positive word-of-mouth advocacy (Bee and Havitz, 2010; Bennett et al., 2009; Funk et al., 2004). Empirical research further demonstrates that fan involvement positively predicts identification levels (Tsiotsou and Alexandris, 2009; Shawn and Philip, 2012), with deeper engagement in a sport or club fostering stronger psychological affiliation (Fisher and Wakefield, 1998). Consequently, highly involved fans are more prone to a halo effect, wherein greater club involvement amplifies their identification with the club’s CSR initiatives. In contrast, low-involvement fans, akin to general consumers, may remain skeptical of the motives behind CSR initiatives despite recognizing the CSR initiatives’ positive valence, necessitating attributional explanations to engage. This leads to the following hypotheses:

H9: For highly involved fans, identification mediates the relationship between perceived CSR and FCIs.

H10: For low-involvement fans, motivational attribution mediates the relationship between perceived CSR and FCIs.

**FIGURE 1:**
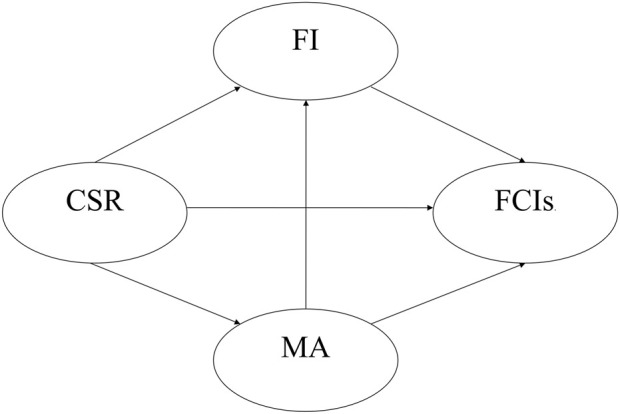
Theoretical model of the study.

The proposed theoretical model is illustrated in Figure 1.

## 3 Methodology

### 3.1 Variable measurement

The measurement of the variables in this study is primarily based on the adaptation of existing international research. Specifically, the perception of CSR among professional football clubs is mainly derived from the works of Walker et al. (2009) and Kim et al. (2016). FI is informed by the research conducted by Wang et al. (2012), while MA is grounded in the theoretical framework proposed by Rifon et al. (2004). Lastly, FCIs is based on the studies by Tzetzis et al. (2014) and Kim and James (2016). The revision procedure comprised three key steps: (1) employing a “two-way translation” method, which involved translating the initial scale of variables into Chinese and subsequently inviting English specialists to translate it back into English for comparative analysis and refinement, resulting in the finalized scale entries; (2) Fan-perceived social responsibility is the core variable in this study. The revision procedure is as follows: first, a content analysis was conducted to collate the social-responsibility information publicly disclosed on the official websites and Sina Weibo accounts of all 21 Chinese Super League clubs. Second, the social-responsibility content was coded and combined with items from existing scales to develop a semi-structured interview protocol; interviews were then carried out with fans to ascertain the content of fan-perceived social responsibility. Third, specific measurement items were generated on the basis of the social-responsibility content perceived by fans.; and (3) administering 188 valid questionnaires to perform a pretest. Item analysis was used to select items with high discriminability, and exploratory factor analysis was employed to identify indicators with high factor loadings. After selecting all items, experts were invited to review and refine them.

The final CSR perception scale for professional football clubs comprises 16 items across 5 dimensions: employee responsibility, fan responsibility, youth responsibility, special population responsibility, and community responsibility. Employee responsibility encompasses care for employees’ personal lives (congratulations on weddings, births, birthdays and other important milestones), concern for players during training and matches (such as when injuries occur), organizing recreational activities for staff, and looking after retired employees (hospital visits, financial assistance, and memorials when they pass away). Fan responsibility includes fan-appreciation initiatives (discounted tickets, football merchandise, dedicated match-day transportation), safeguarding supporters’ rights and interests (ticket handling and financial compensation), and caring for fans (get-well visits for the sick, assistance for the injured or disabled, and general fan welfare). Youth responsibility involves donating welfare items, organizing youth football tournaments, and providing free football coaching in schools. Special population responsibility covers financial aid for people with disabilities, the poor, or the ill; inviting members of special groups to attend matches; and holding tributes during games for victims of major incidents. Community responsibility includes participating in environmental-protection activities, promoting anti-drug campaigns, and fostering the development of amateur football in local communities. Additionally, the scale includes six single-dimension indicators for fan identification, seven single-dimension indicators for motivational attribution, and 11 indicators for consumption intention, encompassing three dimensions: repurchase, word-of-mouth, and licensed merchandise purchase.

For the measurement of involvement, this study adopted the approach used by fitness consumer involvement research (Xu, 2016), fan involvement was assessed through three objective indicators: time spent watching matches, frequency of match attendance, ticket type purchased (e.g., season tickets vs. single-game passes).

### 3.2 Data collection

Our study received approval from the lead author’s Institutional Review Board (IRB) prior to data collection. The formal distribution of questionnaires was conducted through two methods: (1) on-site distribution at Chinese Super League matches, which took place before and during the games, and (2) online distribution to fans’ associations affiliated with the clubs.

In total, 1,115 questionnaires were collected on-site. After excluding incomplete responses and those marked by random answering, 962 valid questionnaires remained. The research team carried out field surveys on 10 September 2017 (Shanghai Shenhua vs. Henan Jianye), 19 September 2017 (Shanghai Shenhua vs. Shandong Luneng) and 23 September 2017 (Shanghai Shenhua vs. Guangzhou R&F) around Shanghai Shenhua FC’s home ground, Hongkou Football Stadium in Shanghai; on 9 September 2017 (Shanghai SIPG vs. Tianjin Yili) and 16 September 2017 (Shanghai SIPG vs. Shanghai Shenhua) around Shanghai SIPG FC’s home ground, the 80,000-seat Shanghai Stadium; and on 28 September 2017 (Jiangsu Suning vs. Tianjin Quanjian) at Jiangsu Suning FC’s home ground, Nanjing Olympic Sports Centre, distributing questionnaires to spectators before and after each match. For the exact number of questionnaires distributed at each club, please refer to Supplementary Material Table S1. For the online distribution, 365 questionnaires were distributed (172 from Shandong Luneng Taishan FC and 193 from Beijing C.H. Guoan FC), and all 365 were deemed valid. Combining both on-site and online distributions, the total number of valid questionnaires was 1,327 (Table 1).

**TABLE 1:** Demographic characterization of the sample.

Category	Option	Sample	Percentage
Gender	Male	803	60.50%
	Female	524	39.50%
Age	Under 18	47	3.54%
	19–25 years	298	22.5%
	26–35 years	527	39.71%
	36–45 years	361	27.20%
	46–55 years	94	7.10%
Monthly Income	No income	346	26.10%
	Below 3,000 RMB	37	2.80%
	3,001-5,000 RMB	121	9.10%
	5,001-8,000 RMB	355	26.80%
	8,001-10,000 RMB	308	23.20%
	Above 10,000 RMB	160	12.10%
Occupation	Student	288	21.70%
	Employee (private and public)	823	62.01%
	Managerial staff	91	6.90%
	Civil servant	31	2.30%
	Freelancer	94	7.10%
Educational Level	Junior high school or below	168	12.70%
	Senior/vocational/technical school or below	389	29.30%
	Bachelor’s/College (including current students)	640	48.20%
	Master’s degree or above	130	9.80%
Years of Spectating	Less than 1 year	320	24.10%
	1–3 years	522	39.30%
	Over 3 years	485	36.50%
Frequency of Spectating	Occasionally (fewer than 5 times)	430	32.40%
	Approximately within 15 times	599	45.10%
	15-30 times	298	22.50%
Ticket Type	Season ticket	810	61.00%
	Single ticket	517	39.00%

### 3.3 Statistical analysis of data

In this study, the questionnaire data were analyzed using SPSS 24.0 and Amos 23.0 statistical software. Discussions were conducted based on the analysis results.

### 3.4 Tables

#### 3.4.1 Reliability

To assess the reliability of the large-sample questionnaire, the study utilized the internal consistency α coefficient. The specific test results are presented in Table 2, where the Cronbach’s α coefficient ranges from 0.879 to 0.909. These values meet the corresponding reliability criteria (high reliability when α ≥ 0.70; acceptable when 0.35 ≤ α ≤ 0.70) (Wu, 2010), indicating that the large-sample questionnaire demonstrates a high level of reliability.

**TABLE 2:** Reliability analysis.

Variable	Dimensions	Cronbach’sα	Composite reliability	AVE	Total Cronbach’s α
CSR	Employee Responsibility	0.787	0.888	0.613	0.909
	Fan Responsibility	0.824			
	Youth Responsibility	0.819			
	Responsibility for special groups	0.792			
	Community responsibility	0.775			
FI	-	-	0.871	0.531	0.879
MA	-	-	0.895	0.551	0.894
FCIs	Repurchase	0.826	0.835	0.628	0.884
	Word-of-mouth	0.828			
	Purchase licensed merchandise	0.804			

#### 3.4.2 Validity analysis

To evaluate the structural validity of the formal questionnaire, the study examined both convergent and discriminant validity. Convergent validity was assessed using validated factor analysis, which involves evaluating two key metrics: the Average Variance Extracted (AVE) and Combined Reliability (CR). According to established criteria, an AVE value of ≥0.5 and a CR value of ≥0.7 indicate acceptable levels of convergent validity (Borsboom et al., 2004). As shown in Table 2, the AVE and CR values for perceived CSR, FI, MA, and FCIs all meet these criteria, confirming that the measurement scales exhibit strong convergent validity.

The discriminant validity of the four core variables (perceived CSR, FI, MA, and FCIs) was evaluated using the results presented in Table 3. Discriminant validity is confirmed when the square root of the AVE for each variable exceeds the correlation coefficients between that variable and all other latent variables. As demonstrated in Table 3, this condition is satisfied for all variables, indicating that the questionnaire possesses robust discriminant validity.

**TABLE 3:** Results of the discriminant validity test.

Variable	CSR	FI	MA	FCIs
CSR	0.783			
FI	0.248	0.729		
MA	0.177	0.271	0.742	
FCIs	0.175	0.452	0.393	0.792

Values on the diagonal indicate the square root of the AVE, of each variable, and values on the off-diagonal indicate the correlation coefficient between the variables.

## 4 Result

This study employs structural equation modeling (SEM) to evaluate the research hypotheses and theoretical framework proposed in the preceding study, which encompass both direct and mediating effects. The direct effect model examines the relationship between the independent variable (CSR) and the dependent variable (FCIs). Two mediation models are examined: Mediation Model 1 assesses the mediating role of FI between fan-perceived CSR and FCIs, whereas Mediation Model 2 incorporates a sequential mediation pathway involving FI and MA to explain the relationship between fan-perceived CSR and FCIs.

### 4.1 Overall model fit

**FIGURE 2:**
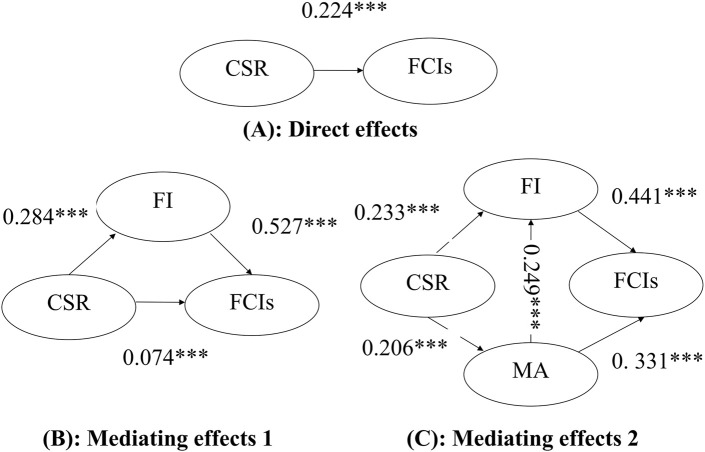
Direct and mediating effects of CSR on FCIs **(A)** Direct effect: CSR → FCIs (β = 0.224). **(B)** Mediating effect 1: CSR → FI (β = 0.284), FI → FCIs (β = 0.527), and the indirect effect CSR → FCIs via FI (β = 0.074). **(C)** Mediating effect 2: CSR → FI (β = 0.233), CSR → MA (β = 0.206), FI → FCIs (β = 0.441), FI → MA (β = 0.249), and MA → FCIs (β = 0.331). All coefficients are significant at p < 0.001.

This study evaluates the theoretical framework proposed in the preceding section by assessing the overall model fit (OMF). OMF is analyzed using absolute fit indices (e.g., RMSEA, SRMR) and incremental fit indices (e.g., CFI, TLI), with detailed criteria and results provided in Table 4. Structural relationships for the direct effect model, Mediation Model 1 (incorporating FI), and Mediation Model 2 (incorporating FI and MA) are illustrated in Figures 2A–C, respectively.

**TABLE 4:** Comparison of direct effect model and mediation effect model.

Model parameter estimation	Direct effect model		Mediation model 1		Mediation model 2		Supported
Hypothesis & path	Standardized coefficient	t	Standardized coefficient	t	Standardized coefficient	t	
CSR→FCIs	0.224***	6.205	0.074*	2.256	0.030	0.969	Yes
CSR→FI	-	-	0.284***	8.098	0.233***	6.888	Yes
CSR→MA	-	-	-	-	0.206***	6.261	Yes
FI→ FCIs	-	-	0.527***	12.996	0.441***	11.643	Yes
MA→ FCIs	-	-	-	-	0.331***	10.113	Yes
MA→FI	-	-	-	-	0.249***	7.842	Yes
R^2^	0.050		0.306		0.403		
Factor loading							
Employee Responsibility →CSR	0.794	-	0.795	-	0.796	-	
Fan Responsibility →CSR	0.837	17.194	0.833	17.205	0.833	17.214	
Youth Responsibility →CSR	0.743	15.979	0.744	16.008	0.744	16.016	
Responsibility for special groups →CSR	0.745	15.747	0.746	15.77	0.744	15.762	
Community responsibility →CSR	0.790	15.680	0.792	15.718	0.793	15.732	
FI	-	-	-	-	-	-	
MA	-	-	-	-	-	-	
Repurchase → FCIs	0.814	-	0.823	-	0.817	-	
Word-of-mouth → FCIs	0.792	16.777	0.786	17.608	0.795	18.018	
Purchase licensed merchandise → FCIs	0.771	15.638	0.768	16.22	0.766	16.566	
Absolute fit indices							
1<χ^2^/df < 3	403.286/315 = 1.280		589.914/484 = 1.219		836.351/726 = 1.152		
RMR<0.05	0.099		0.097		0.095		
SRMR<0.05	0.024		0.024		0.023		
GFI>0.9	0.978		0.973		0.995		
AGFI>0.9	0.974		0.969		0.965		
RMSEA<0.08	0.015		0.013		0.011		
Incremental fit indices							
NFI>0.9	0.974		0.970				
CFI>0.9	0.994		0.994				

As indicated in Table 4, the slightly elevated RMR is due to the large sample size, the root mean square residual (RMR) did not meet established thresholds, necessitating reliance on additional fit indices for comprehensive evaluation (Wu, 2009). Key model fit criteria demonstrated acceptable levels: GFI >0.9, AGFI >0.9, NFI >0.9, CFI >0.9, RMSEA <0.08, and standardized root mean square residual (SRMR) within recommended standards. These results collectively suggest adequate fit for both the direct effect model and the mediation models.

### 4.2 Comparison of direct effect model and mediated effect model

1. The direct effect model analysis reveals a significant positive relationship between fans’ perceived CSR and FCIs, with a standardized path coefficient of 0.224 (t = 6.205 > 1.96). This indicates that heightened perceptions of clubs’ CSR initiatives correlate with stronger FCIs, thereby supporting research hypothesis H1.
2. In mediation model 1, the inclusion of FI as a mediator significantly reduced the standardized path coefficient of perceived CSR on FCIs from 0.224 to 0.074 (t = 2.256 > 1.96). Meanwhile, the model’s explanatory power improved substantially, with the variance explained (R^2^) increasing from 0.050 to 0.306. These results indicate that FI partially mediates the relationship between CSR perceptions and FCIs, thereby validating hypotheses H2, H3, and H4.
3. In mediation model 2, FI and MA fully mediate the effect of fans’ perceived CSR and FCIs, as evidenced by two results: 1) In terms of explanatory power, the mediation effect model 2 has significantly enhanced its explanatory power relative to that of the direct effect model (the value of R^2^ has been enhanced from the original 0.306–0.403); 2) The standardized path coefficient of fans’ perception of CSR on FCIs was 0.030 (t = 0.969 < 1.96), which did not reach a significant level. This indicates that the effect of fans’ perceived CSR on FCIs completely disappears after adding the two mediating variables of FI and MA. In addition, the goodness-of-fit of the mediated effects model 2 improved relative to the direct effects model. Therefore, research hypotheses H5-H7 are valid. Furthermore, based on the path coefficients, the effect of FI on FCIs is greater than the effect of MA on FCIs (standard path coefficient values of 0.441 > 0.331 and t = 11.643 and 10.113), indicating that FI is greater than the effect of MA on FCIs;
4. In mediation model 2, the standardized path coefficient from MA to FI is 0.249 (t = 7.842 > 1.96) indicating that the stronger the fans’ altruistic motivational tendency, the stronger FI with professional football clubs. Therefore, research hypothesis H8 is valid.

### 4.3 The impact of involvement on CSR and FCIs

#### 4.3.1 Clustering of high and low involvement groups

In this paper, we measure fans’ degree of involvement based on three factors: the length of time they watch Chinese Super League soccer matches on-site, the frequency with which they watch matches, and the type of tickets purchased. We then categorize the 1,327 samples using the K-Means Cluster method in cluster analysis. Specifically, we classify the sample into two clusters, and results are presented in Tables 5, 6.

**TABLE 5:** Initial and final clustering centers for fan involvement.

Involvement measurement indicators	Initial clustering center		Final clustering center	
	1	2	1	2
Years of Spectating	3	1	2.64	1.44
Frequency of Spectating	3	1	2.12	1.61
Ticket type	1	2	1.46	1.34

**TABLE 6:** Clustering of high and low involvement groups.

Number of clusters	Percentage (%)	Categories
753	56.7	High Involvement Group
574	43.3	Low Involvement Group

As shown in Table 5, the final cluster centers form two groups. The first cluster has mean scores of 2.64, 2.12, and 1.46, while the second class has measurements of 1.44, 1.61, and 1.34. These measurements indicate that the first class represents the high-involvement group, whereas the second class represents a low-involvement group. There are 753 samples in the high-involvement group and 574 samples in the low-involvement group.

#### 4.3.2 A test of the effect of involvement on the mediation model

As shown in Table 7, several model fit indices exceed 0.9, meeting the recommended threshold. GFI, AGFI, NFI, and CFI all exceed 0.9; RMSEA is below 0.08. SRMR the recommended cutoff, whereas RMR fails to meet its criterion, the slightly elevated RMR is due to the large sample size. Despite RMR not meeting its requirement, the model fit for both high and low involvement groups is acceptable.

**TABLE 7:** SEM model estimation results for high and low involvement groups.

Model parameter estimation hypothesis & path	Low involvement group			Hight involvement group		
	Standardized coefficient	t	Supported	Standardized coefficient	t	Supported
CSR→FCIs	0.003	0.066	No	0.038	0.858	No
CSR→FI	−0.076	−1.440	No	0.483***	8.708	Yes
CSR→MA	0.397***	7.196	Yes	0.066	1.571	No
FI→FCIs	0.418***	7.150	Yes	0.456***	8.500	Yes
MA→FCIs	0.340***	5.992	Yes	0.329***	7.847	Yes
MA→FI	0.335***	6.123	Yes	0.261***	6.620	Yes
Absolute fit indices						
1<χ^2^/df < 3	797.336/726 = 1.098			820.908/726 = 1.131		
RMR<0.05	0.128			0.119		
SRMR<0.05	0.032			0.029		
GFI>0.9	0.936			0.948		
AGFI>0.9	0.928			0.941		
RMSEA<0.08	0.013			0.013		
Incremental fit indices						
NFI>0.9	0.927			0.944		
CFI>0.9	0.993			0.993		

**FIGURE 3:**
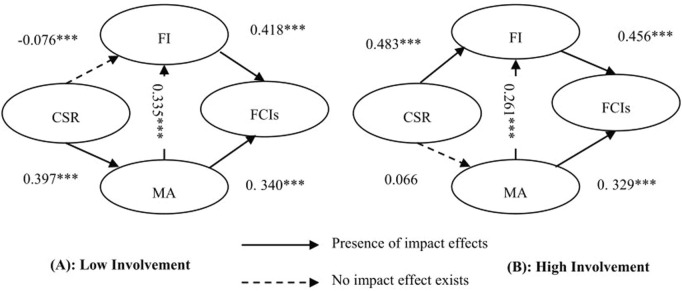
Low involvement and high involvement. **(A)** Low involvement group: only CSR → MA and MA → FCIs paths are significant. **(B)** High involvement group: only CSR → FI and FI → FCIs paths are significant. Solid arrows denote significant effects; dashed arrows denote non-significant effects. Standardized coefficients are shown.

As shown in Table 7 and Figure 3A, the low involvement group demonstrates a significant impact of perceived CSR on MA, with a path coefficient of 0.397 (t = 7.196 > 2.58). However, perceived CSR does not significantly influence FI, as indicated by the path coefficient of −0.076 (t = −1.440 < 2.58). This suggests that in the low involvement fan sample, fans are primarily influenced by the MA of CSR concerning consumption behavior.

Combined with Table 7 and Figure 3B, the high involvement group shows a significant influence of perceived CSR on FI, with a path coefficient of 0.483 (t = 8.708 > 2.58). Conversely, perceived CSR does not significantly impact MA, as evidenced by the path coefficient of 0.006 (t = 1.571 < 2.58). This indicates that in the high involvement group, fans are influenced by perceived CSR and FCIs through the mediating role of FI. Therefore, the research hypotheses H_9_-H_10_ are supported.

## 5 Discussion

This study develops a theoretical model incorporating professional football clubs’ CSR, FI, MA, and willingness to consume behavior. Data were collected through questionnaire distribution and were validated using various mathematical and statistical methods. The main empirical research conclusions are as follows:

First, perceptions of CSR directly and significantly influence FCIs, consistent with findings from prior research (Walker and Kent, 2009; Lacey et al., 2015). This suggests that Chinese Super League fans are inclined to support and acknowledge the club’s CSR actions through their consumption behavior.

Second, the role of CSR in shaping fan’s consumption responses involves not only direct judgment but also an internalized cognitive process mediated by FI and MA. Statistical analyses reveal that FI and MA exert significant mediating effects. Specifically, when these variables are accounted for, the direct effect of CSR on FCIs becomes non-significant, indicating that FI and MA fully mediate the relationship between perceived CSR and consumption behavior.

Third, MA significantly influences FI, with altruistic motives correlating positively with identification levels toward professional football clubs. This underscores MA as a critical determinant of fans’ alignment with clubs’ CSR initiatives.

Fourth, FI exerts a stronger influence on FCIs than MA, highlighting that the effect of clubs’ CSR initiatives on consumption behavior is primarily driven by identification. This phenomenon may stem from two factors. First, cluster analysis revealed that a majority of survey respondents (753 individuals) exhibited high involvement as devoted fans, compared to the 574 individuals with low involvement. Prior research indicates that higher fan involvement correlates with stronger identification (Tsiotsou and Alexandris, 2009; Shawn and Philip, 2012), which in turn amplifies consumption intentions (Wann et al., 2001). Second, the artificial experimental setting of the survey may have encouraged respondents to temporarily endorse CSR initiatives to project an ethical consumer image, rather than reflecting genuine behavioral commitment.

Last, the level of FI moderates the relationship between CSR initiatives and FCIs. For highly involved fans, this relationship is mediated predominantly by FI. Conversely, for fans with low involvement, the effect is driven primarily by MA.

### 5.1 Theoretical implications

Building on extant CSR literature, this study investigates the mediating mechanisms through which fans’ perceptions of CSR influence FCIs, integrating organizational identity theory and MA theory. The research contributes to the field in two key ways:

1. While prior studies on fan consumption responses to CSR in professional sports have focused on European and North American contexts, research in the Chinese context remains limited. Grounded in the Chinese Super League framework, this paper examines how CSR perceptions among fans of Chinese clubs shape consumption intentions, thereby expanding theoretical understanding of CSR in professional sports within the evolving Chinese sports industry.
2. Existing studies on fan consumption responses to clubs’ CSR often treat fans as a homogeneous group, neglecting psychological distinctions between highly involved “devoted fans” and less involved “casual fans.” This study addresses this gap by integrating CSR literature with attribution theory, introducing MA as a novel variable in professional sports CSR research. Furthermore, it examines the moderating role of fan involvement to analyze differential consumption mechanisms between devoted and casual fans in a localized context, thereby advancing theoretical frameworks for CSR in professional sports.

### 5.2 Practical implications

The findings of this study offer practical implications for Chinese professional football clubs’ in implementing CSR. Specifically, they address two critical questions: the necessity of CSR initiatives and strategies for their effective implementation. These insights are summarized in two key recommendations:

First, clubs must strategically integrate CSR as drivers of value creation. Empirical evidence demonstrates that CSR initiatives significantly enhance FCIs. Consequently, club managers should reconceptualize CSR beyond cost-centric or philanthropic frameworks, aligning it with organizational strategies to foster long-term sustainability. This requires embedding CSR into core decision-making processes, transcending traditional financial metrics, and cultivating strategic CSR practices that establish a competitive advantage in the sustainable development of clubs. For example, since 2015 Shanghai Port Football Club has establishing youth-training bases in primary and secondary schools nationwide. Through its “Football Enters the Campus” initiative, the club provides free coaching resources and competition platforms to every partner school, simultaneously fulfilling its social responsibility—addressing the shortage of football resources in remote western rural schools—while tapping into the large pool of campus players to identify and develop promising talents.

Second, clubs must prioritize strategic CSR implementation to prevent perceptions of self-serving motives. While undertaking CSR initiatives to foster fan identification, clubs should avoid overt commercialization that could lead fans to perceive these efforts as performative or profit-driven. Instead, clubs should authentically engage with stakeholders and the community by selecting CSR initiatives aligned with altruistic purposes, thereby strengthening FI and FCIs. Notably, low-involvement fans are particularly sensitive to CSR motive attribution; thus, clubs must exercise caution in both the selection and execution of CSR projects to maintain credibility.

### 5.3 Limitation and future research

The fulfillment of CSR by clubs generates multifaceted value for organizational stakeholders. While this study focuses on CSR’s impact on FCIs, future research could extend this inquiry by examining value creation through other stakeholder lenses. For instance, Employees (e.g., players, coaches, staff): Investigating how CSR initiatives influence employee performance, job satisfaction, and organizational loyalty. Local communities: Analyzing the relationship between CSR efforts and residents’ purchasing behavior or community engagement. Clubs themselves: Exploring CSR’s role in building social capital, enhancing brand equity, or fostering long-term competitive advantages. Such interdisciplinary investigations would provide a holistic understanding of CSR’s strategic potential in professional sports. Moreover, this study operationalized fan involvement solely through behavioral indicators (ticket type, frequency, and duration). While these metrics are objective and readily accessible, they fail to capture attitudinal dimensions such as emotional attachment and cognitive interest—core components of fan involvement. Future research should employ multi-item scales (e.g., the revised Personal Involvement Inventory or the Sport Involvement Scale) to assess affective and cognitive involvement and to cross-validate our findings. Furthermore, the present study does not address how specific types of CSR—namely strategic CSR and instrumental CSR—shape fans’ consumption responses. Future research could disaggregate these CSR categories to examine their distinct impacts on fans’ willingness to spend.

## Data Availability

The original contributions presented in the study are included in the article, further inquiries can be directed to the corresponding authors.

## Abbreviations

CSR, corporate social responsibility; FCIs, fans’ consumption intentions; FI, fan identification; MA, motivational attribution.

## References

[B1] Ashforth B. E. Mael F. (1989). Social identity theory and the organization. Acad. Manag. Rev. 14 (1), 20–39. 10.2307/258189

[B2] Bee C. C. Havitz M. E. (2010). Exploring the relationship between involvement, fan attraction, psychological commitment and behavioural loyalty in a sports spectator context. Int. J. Sports Mark. and Spons. 11 (2), 140–157. 10.1108/IJSMS-11-02-2010-B005

[B3] Bennett G. Cunningham G. Dees W. (2009). The role of involvement in sports and sport spectatorship in sponsor’s brand use: the case of Mountain Dew and action sports sponsorship. Sport Mark. Q. 18 (1), 14–24. 10.1080/02614360903254366

[B4] Borsboom D. Mellenbergh G. J. van Heerden J. (2004). The concept of validity. Psychol. Rev. 111 (4), 1061–1071. 10.1037/0033-295X.111.4.1061 15482073

[B5] Breitbarth T. Harris P. (2008). The role of corporate social responsibility in the football business: towards the development of a conceptual model. Eur. Sport Manag. Q. 8 (2), 179–206. 10.1080/16184740802024484

[B6] Cialdini R. B. Borden R. J. Thorne A. Walker M. R. Freeman S. Sloan L. R. (1976). Basking in reflected glory: three (football) field studies. J. Personality Soc. Psychol. 34 (3), 366–375. 10.1037/0022-3514.34.3.366

[B7] Deng X. Long X. (2017). Corporate social responsibility, corporate evaluation and consumer response. J. Zhongnan Univ. Econ. Law (5), 126–136. 10.3969/j.issn.1003-5230.2017.05.014

[B8] Deng X. Zhang T. (2016). Research on the impact of corporate social responsibility on consumer purchase intention. Chin. J. Manag. 13 (7), 1019–1027. 10.3969/j.issn.1672-884X.2016.07.008

[B9] Ellen P. S. Webb D. J. Mohr L. A. (2006). Building corporate associations: consumer attributions for corporate socially responsible programs. J. Acad. Mark. Sci. 34 (2), 142–157. 10.1177/0092070305284976

[B10] Fisher R. J. Wakefield K. (1998). Factors leading to group identification: a field study of winners and losers. Psychol. and Mark. 15 (1), 23–40. 10.1002/(SICI)1520-6793(199801)15:1<23::AID-MAR3>3.0.CO;2-3

[B11] Funk D. C. Ridinger L. L. Moorman A. M. (2004). Exploring origins of involvement: understanding the relationship between consumer motives and involvement with professional sport teams. Leis. Sci. 26 (1), 35–61. 10.1080/01490400490272440

[B12] Gao Y. (2009). Corporate social performance in China: evidence from large companies. J. Bus. Ethics 89 (1), 23–35. 10.1007/s10551-008-9982-y

[B13] Gladden J. M. Funk D. C. (2001). Understanding brand loyalty in professional sport: examining the link between brand associations and brand loyalty. International *Journal of Sports Marketing and Sponsorship* . 3 (1), 67–94. 10.1108/IJSMS-03-01-2001-B006

[B14] Groza M. D. Pronschinske M. R. Walker M. (2011). Perceived organizational motives and consumer responses to proactive and reactive CSR. J. Bus. Ethics 102 (4), 639–652. 10.1007/s10551-011-0834-9

[B15] Jiang L. Shi M. (2017). The relationship between corporate donation based on emotional perception and consumer response. Consum. Econ. 33 (4), 52–58. 10.3969/j.issn.1007-5682.2017.04.007

[B16] Jin L. (2006). An empirical study on the evaluation index system of corporate social responsibility movement. China Ind. Econ. (6), 114–120. 10.19581/j.cnki.ciejournal.2006.06.012

[B17] Kelley H. H. (1973). The processes of causal attribution. Am. Psychol. 28 (2), 107–128. 10.1037/h0034225

[B18] Kim M. S. James J. D. (2016). The theory of planned behaviour and intention of purchase sport team licensed merchandise. Sport, Business and Management. Int. J. 6 (2), 228–243. 10.1108/SBM-02-2015-0004

[B19] Kim I.-G. Kim S. Lee Y. K. Kim J. Y. (2016). Analysis of corporate social responsibility (CSR) activity types of Korean professional sports team: application of coorientation model. Indian J. Sci. Technol. 9 (25), 1–11. 10.17485/ijst/2016/v9i25/97240

[B20] Klein J. Dawar N. (2004). Corporate social responsibility and consumers' attributions and brand evaluations in a product-harm crisis. Int. J. Res. Mark. 21 (3), 203–217. 10.1016/j.ijresmar.2003.12.003

[B21] Kou Y. (2008). The impact of corporate social responsibility behavior on consumer response: the role of consumer expectations and perceived motives [*Doctoral dissertation, Southwestern University of Finance and Economics*]. CNKI. 10.27222/d.cnki.gcxju.2008.000012

[B22] Lacey R. Kennett-Hensel P. A. (2016). How expectations and perceptions of corporate social responsibility impact NBA fan relationships. Sport Mark. Q. 25 (1), 21–33. 10.32731/SMQ.251.032016.03

[B23] Lacey R. Kennett-Hensel P. A. Manolis C. (2015). Is corporate social responsibility a motivator or hygiene factor? Insights into its bivalent nature. J. Acad. Mark. Sci. 43 (3), 315–332. 10.1007/s11747-014-0390-9

[B24] Lock D. Taylor T. Funk D. Darcy S. (2012). Exploring the development of team identification. J. Sport Manag. 26 (4), 283–294. 10.1123/jsm.26.4.283

[B25] Ma L. (2011). Research on the influence mechanism of corporate social responsibility on consumer purchase intention. Mod. Manag. Sci. (5), 120–126. 10.3969/j.issn.1007-368X.2011.05.030

[B26] Mason T. (2001). Nike and DoE tackle school bullying. Marketing 17.

[B27] Mohr L. A. Webb D. J. (2005). The effects of corporate social responsibility and price on consumer responses. J. Consumer Aff. 39 (1), 121–147. 10.1111/j.1745-6606.2005.00006.x

[B28] Rifon N. J. Choi S. M. Trimble C. S. Li H. (2004). Congruence effects in sponsorship: the mediating role of sponsor credibility and consumer attributions of sponsor motive. J. Advert. 33 (1), 30–42. 10.1080/00913367.2004.10639151

[B29] Scheinbaum A. C. Lacey R. (2015). Event social responsibility: a note to improve outcomes for sponsors and events. J. Bus. Res. 68 (9), 1982–1986. 10.1016/j.jbusres.2015.01.008

[B30] Shank M. D. Beasley F. M. Gordon J. R. (1998). Fan or fanatic: refining a measure of sports involvement. J. Sport Behav. 21 (4), 435–444. 10.1123/jsb.21.4.435

[B47] Shawn S. Philip J. R. (2012). The influence of involvement, following sport and fan identification on fan loyalty: An Australian perspective. Int. J. Sports Mark. Spons. 13 (3), 57–71. 10.1108/IJSMS-13-03-2012-B006

[B31] Sherif M. Cantril H. (1947). The psychology of ego-involvements. John Wiley and Sons.

[B32] Sutton W. A. McDonald M. A. Milne G. R. Cimperman J. (1997). Creating and fostering fan identification in professional sports. Sport Mark. Q. 6 (1), 15–22. 10.1080/10696679.1997.11501737

[B33] Taylor S. F. Rice P. J. (2012). The influence of involvement, following sport and fan identification on fan loyalty: an Australian perspective. Int. J. Sports Mark. Spons. 13 (3), 57–71. 10.1108/IJSMS-13-03-2012-B006

[B34] Tsiotsou R. Alexandris K. (2009). Delineating the outcomes of sponsorship: sponsor image, word of mouth, and purchase intentions. Int. J. Retail and Distribution Manag. 37 (4), 358–369. 10.1108/09590550910948583

[B35] Tzetzis G. Alexandris K. Kapsampeli S. (2014). Predicting visitors’ satisfaction and behavioral intentions from service quality in the context of a small-scale outdoor sport event. Int. J. Event Festiv. Manag. 5 (1), 4–21. 10.1108/IJEFM-09-2012-0026

[B36] Underwood R. Bond E. Baer R. (2001). Building service brands via social identity: lessons from the sports marketplace. J. Mark. Theory Pract. 9 (1), 1–13. 10.1080/10696679.2001.11501883

[B37] Vlachos P. A. Epitropaki O. Panagopoulos N. G. Rapp A. A. (2013). Causal attributions and employee reactions to corporate social responsibility. Industrial Organ. Psychol. 6 (4), 334–337. 10.1111/iops.12077

[B38] Walker M. Kent A. (2009). Do fans care? Assessing the influence of corporate social responsibility on consumer attitudes in the sport industry. J. Sport Manag. 23 (6), 743–769. 10.1123/jsm.23.6.743

[B39] Walker M. Heere B. Parent M. M. Drane D. (2010). Social responsibility and the olympic games: the mediating role of consumer attributions. J. Bus. Ethics 95 (4), 659–680. 10.1007/s10551-010-0445-x

[B40] Wang M. C.-H. Chen J.-S. Chin T.-S. (2012). The purchasing impact of fan identification and sports sponsorship. Mark. Intell. and Plan. 30 (5), 553–566. 10.1108/02634501211251025

[B41] Wann D. L. Melnick M. J. Russell G. W. Pease D. G. (2001). Sport fans: *The psychology and social impact of spectators* . London, United Kingdom: Routledge.

[B42] Weiner B. (1989). An attributional theory of motivation and emotion. 10.1007/978-1-4612-3618-4 3903815

[B43] Weiner B. (1992). An attribution theory of achievement motivation and emotion. Psychol. Rev. 99 (4), 548–573. 10.1037/0033-295X.99.4.548 3903815

[B44] Wu M. L. (2009). Structural equation modeling: operation and application of AMOS. Chongqing, China: Chongqing University Press.

[B45] Wu M. L. (2010). Questionnaire statistical analysis practice: SPSS operation and application. Chongqing, China: Chongqing University Press.

[B46] Xu K. (2016). Research on improving service quality of commercial fitness clubs from the perspective of service recovery. CNKI. 10.7666/d.D01129617

